# Supplementary figures and images for: Evaluation of the Inhibitory Potential of Synthetic Peptides Homologous to CDR3 Regions of a Monoclonal Antibody against Bothropic Venom Serine Proteases
Source: Int J Mol Sci. 2024 May 9;25(10):5181. doi: 10.3390/ijms25105181 (PMC11121450; doi:10.3390/ijms25105181)

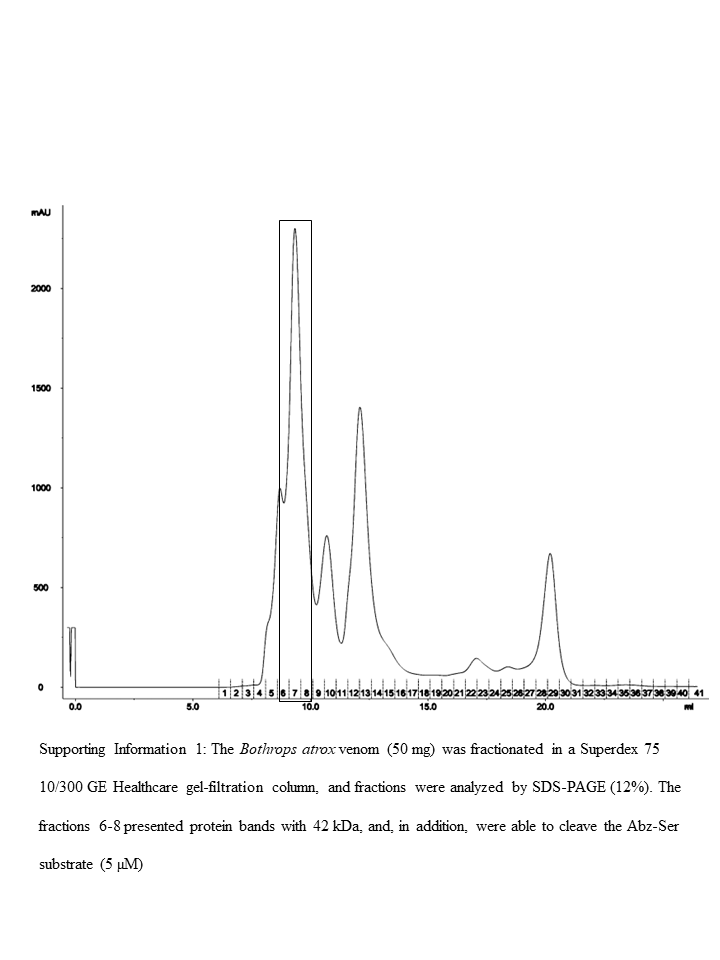

Supplement: Supplementary file 1 [file ijms-25-05181-s001.zip › Figure S1.tif]

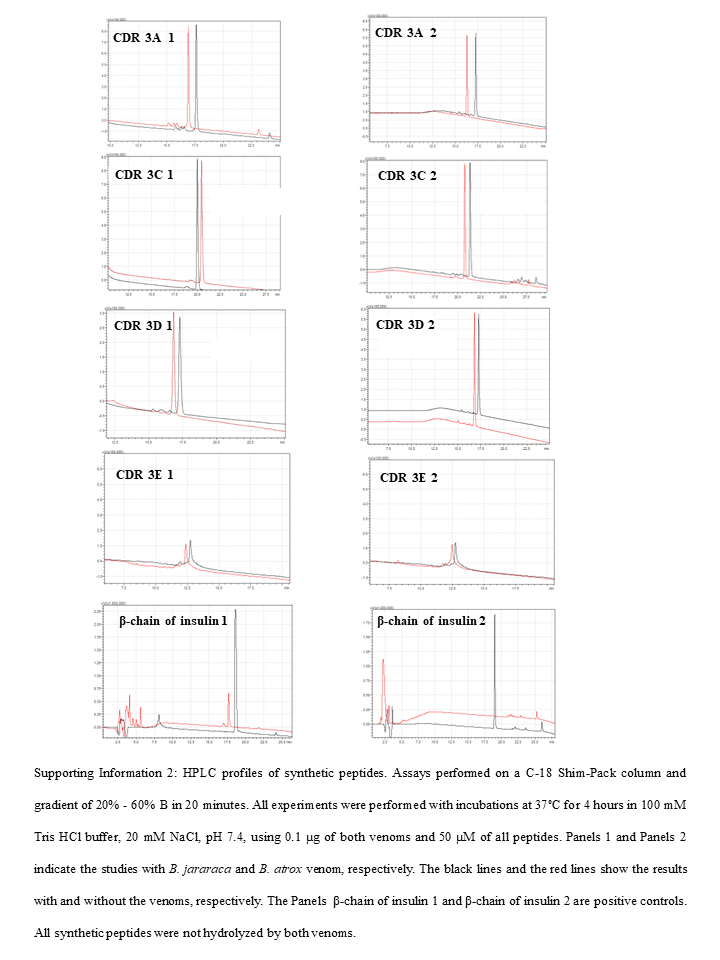

Supplement: Supplementary file 1 [file ijms-25-05181-s001.zip › Figure S2.tif]

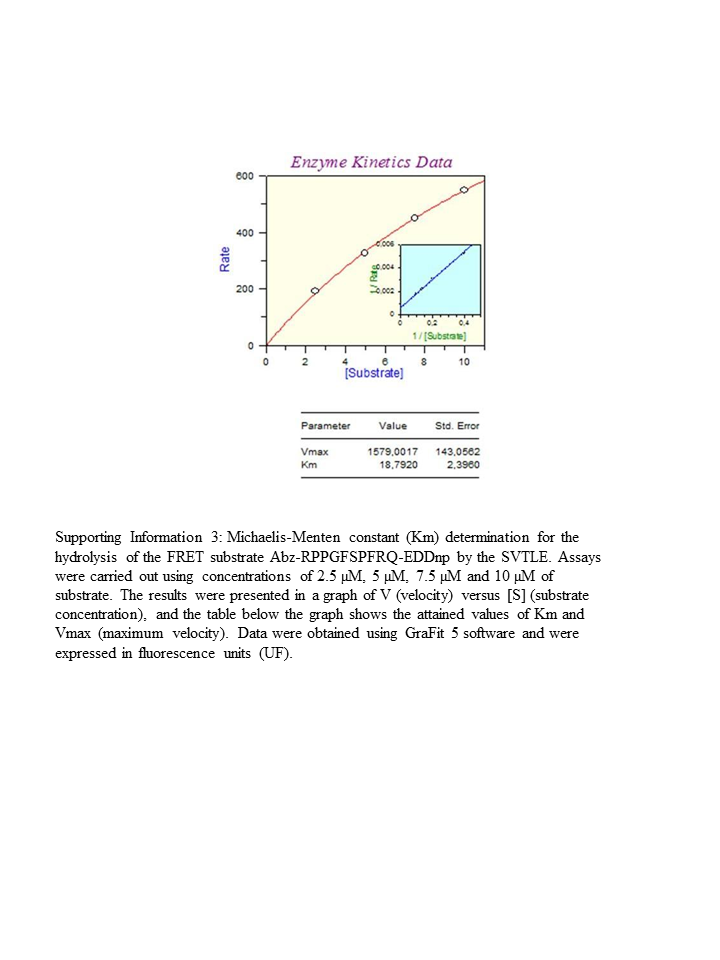

Supplement: Supplementary file 1 [file ijms-25-05181-s001.zip › Figure S3.tif]
